# Clinical Significance of APOE4 Genotyping: Potential for Personalized Therapy and Early Diagnosis of Alzheimer’s Disease

**DOI:** 10.3390/jcm14176047

**Published:** 2025-08-26

**Authors:** Jelena Rajič Bumber, Valentino Rački, Silvestar Mežnarić, Gordana Pelčić, Jasenka Mršić-Pelčić

**Affiliations:** 1 Department of Basic and Clinical Pharmacology and Toxicology, Faculty of Medicine, University of Rijeka, 51000 Rijeka, Croatia; silvestar.meznaric@medri.uniri.hr (S.M.); jasenka.mrsic.pelcic@medri.uniri.hr (J.M.-P.); 2 Department of Neurology, Faculty of Medicine, University of Rijeka, 51000 Rijeka, Croatia; valentino.racki@medri.uniri.hr; 3 Department of Neurology, Clinical Hospital Centre Rijeka, 51000 Rijeka, Croatia; 4 Health Care Center of Primorsko-Goranska County, Rijeka, 51000 Rijeka, Croatia; gordana.pelcic@medri.uniri.hr; 5 Department of Social Sciences and Medical Humanities, School of Medicine, University of Rijeka, 51000 Rijeka, Croatia

**Keywords:** *APOE4*, Alzheimer’s disease, genotyping, precision medicine

## Abstract

Apolipoprotein E (APOE) remains the most robust and widely replicated genetic risk factor for late-onset Alzheimer’s disease (AD) susceptibility, with the ε4 allele (*APOE4*) demonstrating profound associations with accelerated symptom manifestation, enhanced disease trajectory, and modified therapeutic responsiveness. This comprehensive review synthesizes contemporary evidence regarding the clinical utility of *APOE4* genotyping, emphasizing its integration into personalized therapeutic frameworks and early diagnostic paradigms. The APOE4 variant exerts pathogenic influence through impaired amyloid-β clearance, enhanced tau pathology, and compromised neuronal repair mechanisms that alter disease phenotype. We systematically examine available genotyping methodologies, encompassing polymerase chain reaction (PCR) and next-generation sequencing (NGS) platforms, and evaluate their practical implementation within clinical environments. Recent investigations demonstrate that *APOE4* status profoundly influences therapeutic efficacy, particularly with anti-amyloid interventions such as lecanemab, where carriers exhibit enhanced treatment response alongside increased adverse event susceptibility. Emerging gene therapeutic approaches show promise in mitigating *APOE4*-associated risks through targeted molecular interventions. The integration of *APOE4* genotyping with fluid biomarkers and neuroimaging techniques enables refined patient stratification and enhanced diagnostic precision, facilitating earlier intervention windows that optimize therapeutic outcomes before irreversible neuronal damage occurs. This review underscores *APOE4* testing as a transformative component of precision medicine in AD management, emphasizing its contribution to diagnostic refinement, clinical decision support, and targeted therapeutic interventions.

## 1. Introduction

Alzheimer’s disease (AD) is the most common cause of dementia worldwide, accounting for approximately 60–70% of cases, and is characterized by a progressive decline in cognitive functions, memory impairment, and deterioration in daily functioning [1]. Globally, over 55 million people live with dementia, a figure expected to rise to 139 million by 2050 due to aging populations [2]. In the United States, more than 6 million individuals are affected, with healthcare costs exceeding USD 300 billion annually, expected to almost triple in the coming decades [3]. Beyond its economic burden, AD profoundly impacts patients, families, and caregivers emotionally and socially, underscoring the necessity for improved diagnostic, therapeutic, and preventive measures [4,5,6].

Clinically, AD develops insidiously, most commonly after the age of 65. Early symptoms include short-term memory loss, subtle executive dysfunction, and language difficulties, often accompanied by mood changes such as anxiety or agitation. As the disease progresses, patients experience severe neurocognitive deficits, including disorientation, loss of independence, behavioral changes, hallucinations, and increased confusion. These symptoms reflect the deterioration of brain regions vital for memory and cognition, like the hippocampus [6,7,8].

Neuropathologically, AD is defined by the accumulation of extracellular amyloid-beta (Aβ) plaques and intracellular neurofibrillary tangles of hyperphosphorylated tau protein. These hallmark features, though central to diagnosis and therapeutic research, are part of a multifactorial disease etiology involving genetic, environmental, and lifestyle factors [9,10].

Genetics plays an important role in AD risk. Although pathogenic variants in the amyloid precursor protein (APP), presenilin 1 (PSEN1), and presenilin 2 (PSEN2) genes account for less than 1% of all AD cases, nearly all individuals carrying such mutations will eventually develop the disease. These variants underlie early-onset autosomal dominant AD and are also implicated in AD associated with Down syndrome, where individuals, due to trisomy 21, carry three copies of the APP gene [11,12,13]. Symptom onset in these variants typically occurs between 40 and 60 years of age, and clinical, pathological, and biomarker alterations follow a relatively predictable trajectory [14]. The vast majority of AD cases, however, are sporadic or late-onset forms, with over 75 genetic loci associated with increased disease risk [15,16]. Among these, the apolipoprotein E (APOE) gene, particularly the ε4 allele (*APOE4*), remains the strongest and most consistent genetic risk factor identified to date. Over the past three decades, roughly ten thousand scientific publications have examined various aspects of the relationship between *APOE* and AD [17,18,19,20]. Recent evidence suggests that the predictability of symptom onset and the temporal sequence of biomarker alterations in *APOE4* homozygotes closely parallel those observed in autosomal dominant AD and Down syndrome. On this basis, some researchers now propose that *APOE4* homozygosity constitutes a distinct genetic form of AD, positioning it closer to a deterministic genotype than a conventional risk factor [21]. However, this interpretation remains a matter of ongoing scientific debate, and while *APOE4/E4* is associated with substantially elevated risk and a relatively predictable biomarker trajectory, it is not deterministic to the same extent as pathogenic variants in *APP*, *PSEN1*, or *PSEN2*. Accordingly, proposals to classify *APOE4* homozygosity as a genetically defined susceptibility state for AD should be made with caution, particularly in view of the potential psychological and ethical implications for patients.

Given the pivotal role of *APOE4* in AD, genotyping for *APOE* status has become integral to many research and clinical trials, informing risk stratification, prognosis, and therapeutic responses. For example, responses to anti-amyloid monoclonal antibodies like lecanemab, donanemab, and aducanumab appear modulated by *APOE* genotype, prompting the development of gene-based and lipid-modulating treatments targeting APOE4-related pathological pathways [22,23]. However, routine *APOE* genotyping in clinical practice remains limited due to ethical and logistical challenges. Advances in minimally invasive genotyping techniques, such as polymerase chain reaction (PCR) and next-generation sequencing (NGS) from peripheral blood or saliva, alongside biomarker and imaging innovations, offer promising avenues for earlier diagnosis and personalized medicine in AD management [4,24].

This review thus focuses on the clinical relevance of *APOE4* genotyping, discussing current techniques, the impact of APOE4 on therapy and biomarker interpretation, and potential personalized treatment approaches, aiming to improve early detection and targeted intervention in AD.

## 2. *APOE4*: Genetic Basis and Mechanisms of Action

### 2.1. Chromosomal Localization and Physiological Role of the APOE Gene

**Figure 1:**
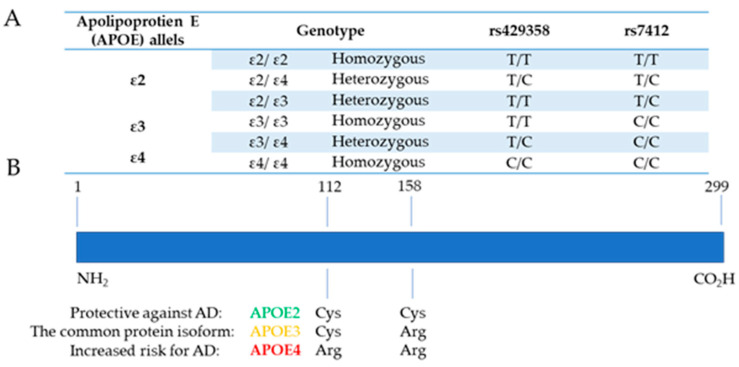
Molecular basis of *APOE* genetic variants and corresponding protein isoforms. (**A**) A schematic overview of the three major *APOE* alleles (ε2, ε3, and ε4) and their genotype combinations, defined by two single-nucleotide variants (SNVs): *rs429358* and *rs7412*. These SNVs lead to amino acid substitutions at positions 112 and 158, resulting in distinct APOE protein isoforms. (**B**) A structural representation of the APOE protein, highlighting isoform-specific amino acid differences. APOE3 is the most prevalent isoform in the general population and is considered risk-neutral. APOE2 is associated with a protective effect against Alzheimer’s disease (AD), while APOE4 confers significantly increased risk for late-onset AD. Isoform-specific structural differences influence lipid-binding capacity, receptor affinity, and functional roles in neurodegeneration.

The *APOE* gene is located on the long arm of chromosome 19 (19q13.32), comprises four exons and three introns, covering 3597 base pairs [25,26]. It encodes a 34-kDa APOE protein of 299 amino acids. This protein, initially described in the early 1970s, plays a central role in the transport and metabolism of plasma cholesterol and triglycerides, and is also critically involved in central nervous system (CNS) processes, including injury response and repair [27]. The human *APOE* gene is polymorphic and exists in three common allelic forms: *APOE* ε2, ε3, and ε4. Its inheritance is codominant, resulting in six genotypes: three homozygous genotypes (ε2/ε2, ε3/ε3, and ε4/ε4) and three heterozygous (ε2/ε3, ε2/ε4, and ε3/ε4). *APOE* ε3 allele (*APOE3*) represents the reference, normal function allele. These alleles result from non-synonymous exonic single-nucleotide variants (SNVs; formerly single-nucleotide polymorphisms, SNPs) at two closely located loci that are labeled as rs429358 (C>T) and rs7412 (C>T) [28] (Figure 1A).

These variants cause amino acid substitutions at positions 112 and 158, respectively, in the APOE protein [29,30]. The haplotype combination of these two SNVs determines the three major APOE protein isoforms. The most common isoform, APOE3, contains a cysteine at position 112 and an arginine at position 158. The least common isoform, APOE2, has cysteine residues at both positions, while APOE4 contains arginine at both sites (Figure 1B). The presence of arginine at position 112 and cysteine at position 158 is extremely rare and defines the APOE1 (also referred to as APOE3r) isoform [31]. Although the three common isoforms differ by only one or two amino acids, these differences have been shown to influence the structural conformation of the APOE protein by promoting domain interaction, thereby altering its biophysical properties and function. In APOE4, the N-terminal and C-terminal domains, usually spatially separated in APOE2 and APOE3, are brought into proximity through the formation of a salt bridge [32,33]. The N-terminal part of the protein interacts with receptor proteins that are members of the low-density lipoprotein receptor family, while the C-terminal part interacts with lipids. In addition, APOE also binds directly to Aβ peptides. These structural differences among APOE isoforms result in distinct binding affinities to APOE receptors involved in lipid transport and diverse cellular processes, including monomeric Aβ clearance from the brain and effects on neural regeneration [34,35].

### 2.2. APOE Isoforms and Alzheimer’s Disease: Differential Risk Profiles of APOE2, APOE3, and APOE4

The *APOE4* is present in about 24% of individuals who carry at least one ε4 allele, with considerable variation across ancestral groups [18,36]. However, the prevalence of *APOE4* is markedly higher among individuals with AD, being detected in 55–75% of those with AD dementia, around 57% of individuals with prodromal AD, and approximately 55% of those in the preclinical stage of the disease [37]. *APOE*-related risk for AD follows *APOE* ε2 < *APOE* ε3 < *APOE* ε4 for Caucasians. *APOE* ε4 frequencies in patients with other amyloid-forming diseases are comparable to those in healthy controls, despite the consistent presence of APOE within amyloid plaques across these conditions [38]. *APOE* ε2 allele (*APOE2*) appears to be protective against AD, demonstrated across decades of research and reaffirmed in recent genome-wide association studies and meta-analyses [39,40,41,42]. In cellular studies, *APOE*-null cells exhibited phenotypes broadly similar to those of cells expressing the *APOE* ε3/ε3 genotype [43]. The risk of developing AD increases in a dose-dependent manner with the number of *APOE* ε4 alleles, while the age at onset correspondingly decreases [18]. General estimates indicate that individuals carrying a single *APOE* ε4 allele have a 2-to-4-fold increased risk of AD, whereas homozygous carriers exhibit an approximately 8-to-12-fold elevated risk. The magnitude of this risk is modulated by factors such as ancestral background and sex [18,44,45]. In addition to increased risk, *APOE4* is associated with an earlier clinical manifestation of AD, with carriers developing symptoms on average 12 years earlier than non-carriers [46]. The *APOE4* exhibits semidominant inheritance concerning AD risk, whereby homozygous individuals face a lifetime risk of dementia approaching 60% by age 85, substantially exceeding that of heterozygous carriers or noncarriers. Nevertheless, about 40% remain unaffected, reflecting incomplete penetrance influenced by modifying genetic loci, vascular and metabolic comorbidities, and environmental exposures. This dose-dependent effect is comparable in magnitude to the impact of *BRCA1* mutations on hereditary breast cancer susceptibility [19].

### 2.3. Pathogenic Mechanisms Underlying APOE4-Associated Disease Risk

APOE4 has been linked with a broad range of pathobiological effects relevant to AD. These include impaired neurite outgrowth, cytoskeletal disruption and tau hyperphosphorylation, mitochondrial dysfunction in neurons, impaired synaptogenesis, increased Aβ production, altered Aβ clearance and deposition, lysosomal leakage, and enhanced neuronal apoptosis [26,47].

Recent studies employing advanced research tools and experimental models have demonstrated that the impact of APOE4 on AD pathology is influenced by both its cellular origin and expression levels [48]. Multiple cell types within the mammalian CNS, under different conditions, are capable of producing and secreting APOE, mainly astrocytes and microglia but also neurons, oligodendrocytes, and vascular cells. In peripheral tissues, APOE is synthesized primarily by the hepatocytes (>90%), with additional expression observed in the adrenal glands and macrophages [49,50]. Due to the inability of APOE to efficiently cross the blood–brain barrier (BBB), the peripheral and central pools of APOE are generally regarded as functionally and biochemically distinct [51,52,53].

*APOE4* neurons display elevated Aβ secretion and phospho-tau (p-tau) accumulation compared to *APOE3* neurons, effects that are reversed by *APOE* knockout and restored upon *APOE4* reintroduction [43]. Moreover, transcriptomic and cytokine analyses demonstrate that *APOE4* drives microglial polarization toward a proinflammatory state, in contrast to the more homeostatic and neuroprotective phenotype observed in *APOE2*-expressing microglia [54]. Furthermore, *APOE4* carriers, both in human AD cohorts and AD mouse models, exhibit oligodendrocyte dysfunction, impaired myelination, and BBB disruption [55]. In addition to these hallmark pathologies, APOE4 also drives lipid and cholesterol accumulation and disrupts cell–cell communication across various cell types [56,57,58].

A recent proteomic analysis of CSF samples revealed elevated APOE4 protein levels in individuals with AD-related cognitive impairment or AD biomarker positivity compared to controls. Furthermore, integration of proteomic, genetic, and mediation analyses within the same study demonstrated that the *APOE* ε4 allele is associated with increased APOE4 protein levels, which, in turn, mediate elevated risk for AD [59]. Based on cumulative evidence from that and numerous other human and animal studies, researchers involved in the National Institute on Aging’s Alzheimer’s Disease Sequencing Project reached a consensus supporting the view that lowering APOE4 levels constitutes a rational therapeutic strategy, particularly in individuals of African and European ancestry carrying the *APOE* ε4 allele [60].

Although *APOE* is most extensively studied in the context of AD, it is not specific to AD pathology. The gene has also been associated with poorer clinical outcomes following traumatic brain injury [61], as well as other neurological disorders in which the BBB or the immune system play a substantial role [62,63], but its precise role outside of AD remains incompletely understood and continues to be an active area of research. In addition, APOE has broader physiological significance beyond the nervous system, and it has been strongly implicated in cardiovascular diseases. The six *APOE* genotypes show a stepwise, parametric relationship with plasma levels of APOE and high-density lipoprotein cholesterol, which progressively decrease across the genotype spectrum (ε2 > ε3 > ε4), while low-density lipoprotein cholesterol levels exhibit the opposite. This lipid profile gradient is mirrored in cardiovascular risk, with coronary artery disease and myocardial infarction risk being the lowest among *APOE* ε2 carriers, intermediate in ε3 carriers, and the highest among ε4 carriers [64,65,66]. *APOE4*-positive individuals exhibit an approximately 30% increased risk of ischemic stroke [67]. It remains uncertain whether this elevated risk is solely attributable to ε4-associated hypercholesterolemia and coronary artery disease, or whether additional brain-specific mechanisms are involved, such as the role of APOE in astrocytes and pericytes at the BBB [68].

## 3. Diagnostic Dynamics and Methodology of *APOE4* Genotyping

The methodological approach to *APOE4* genotyping is based on rigorous laboratory protocols, method validation, quality control, and test standardization, as well as ethical and societal frameworks. The future of the field increasingly incorporates advanced technologies and artificial intelligence [69,70]. This paragraph provides a structured overview of the methodology, the benefits, and the challenges of routine *APOE4* status determination in the context of therapy personalization.

The search for an early diagnostic method for AD involving the *APOE4* genotype began back in 1993 with work by Saunders et al. [38]. In the late 2000s, the development of high-throughput genomic approaches enabled the discovery of additional genetic risk factors for AD upon the earlier identification of three major *APOE* alleles. Over the past 15 years, approximately 75 loci that represent risk factors for AD progression have been found thanks to genome-wide association studies and sequencing projects [16,17,71,72,73].

### 3.1. PCR Technologies Without Fluorescent Detection Systems

The first standard approach developed in the 1990s is PCR—restriction fragment length polymorphism (PCR-RFLP). The method includes amplification of a specific *APOE* gene segment followed by restriction digestion, which allows for differentiation of alleles based on different fragmentation patterns. This traditional approach, while reliable and widely adopted as a reference standard, requires post-PCR processing steps, including gel electrophoresis for fragment analysis, making it time-consuming and labor-intensive compared to newer real-time PCR methods. The method has notable limitations, including complex electrophoretic patterns that can result from partial enzymatic digestion, potential for ambiguous results due to incomplete restriction, and the requirement for specialized gel documentation systems. Additionally, RFLP analysis may fail to detect certain polymorphisms, as demonstrated by cases where previously unknown (novel) sequence changes within the DNA segment encoding codon 158 were not recognized by enzyme digestion but were successfully identified by more sensitive fluorescence-based methods, highlighting the superior resolution and reliability of newer genotyping approaches [74,75].

Amplification refractory mutation system PCR (ARMS-PCR) is a molecular method that utilizes allele-specific primers for selective DNA amplification, enabling the detection of known point mutations or polymorphisms without requiring restriction enzymes. The method is based on the principle that a primer with a mismatched base at the 3’ end will not permit amplification, while perfect matching results in successful amplification. The advantages of ARMS-PCR include simplicity of execution, rapid results, low reagent costs, multiplexing capability (simultaneous detection of multiple variants), and independence from expensive real-time PCR equipment or restriction enzymes. This method was particularly suitable for large population studies due to its cost-effectiveness and automation potential. However, ARMS-PCR has notable limitations, including the requirement for precise primer design, sensitivity to DNA sample quality, potential for non-specific amplification under suboptimal conditions, and inability to detect novel, unknown mutations. Additionally, result interpretation can be complex in heterozygotes due to varying band intensities. Despite these limitations, the success rate in *APOE* detection has proven exceptionally high, with studies reporting 94–100% accuracy compared to Sanger sequencing as the gold standard. The tetra-ARMS PCR variant enables simultaneous detection of both crucial *APOE* polymorphisms in a single reaction, making it superior to the PCR-RFLP method.

Sequence-specific primer PCR (SSP-PCR) represents a genotyping approach that utilizes haplotype-specific primers to identify *APOE* allelic combinations through differential amplification patterns directly. The molecular basis relies on the principle that primers with 3’-end nucleotide variations specific for polymorphic positions will only amplify when there is perfect complementarity with the target sequence [76]. The method employs two forward primers targeting position 2059 variants (Cys112 vs. Arg112) and two reverse primers specific for position 2197 variants (Cys158 vs. Arg158), combined in three reaction mixtures: “Primer Mix E2” for Cys112/Cys158, “Primer Mix E3” for Cys112/Arg158, and “Primer Mix E4” for Arg112/Arg158 combinations. Advantages of SSP-PCR methodology include its cost-effectiveness due to non-labeled primer usage, rapid turnaround time, compatibility with standard PCR equipment, and the ability to determine cis/trans chromosomal orientation of alleles simultaneously [77]. The method demonstrates exceptional reliability with 100% concordance compared to Sanger sequencing validation and can accommodate various DNA extraction methods and sample qualities, making it highly accessible for routine diagnostic laboratories. Additionally, SSP-PCR enables high-throughput processing of multiple samples simultaneously while maintaining accuracy and reproducibility. Primary limitations encompass the requirement for precise primer design and optimization, sensitivity to PCR conditions such as annealing temperatures and primer concentrations, and potential for non-specific amplification, requiring careful validation with control primers [76]. The method also necessitates gel electrophoresis, which limits throughput compared to real-time approaches, and incomplete amplification may occur with degraded DNA samples. While SSP-PCR represented a significant advancement over PCR-RFLP methods, which suffered from incomplete restriction enzyme digestion and time-consuming procedures, the molecular diagnostics field demanded even greater automation, higher throughput capabilities, and reduced hands-on time.

### 3.2. Real-Time PCR Technologies with Fluorescent Detection

The emergence of real-time PCR technologies with fluorescent detection systems offered closed-tube analysis, eliminating post-PCR processing steps and reducing contamination risks that were associated with earlier-mentioned gel-based methods. These technological advances ultimately led to the development of TaqMan probe-based assays and other real-time approaches that could provide faster results with minimal manual intervention, though at considerably higher reagent costs. While methods such as ARMS-PCR and SSP-PCR are based on selective DNA amplification using allele-specific primers, which enable the detection of known point mutations or polymorphisms, RT-PCR with melting curve analysis enables real-time monitoring of amplification and allele differentiation based on fluorescent signal changes during denaturation or fluorescent probe interactions.

Real-time PCR with melting curve analysis represents a cost-effective method for *APOE* genotyping that utilizes SYBR Green fluorescent dye to monitor DNA amplification and subsequent melting curve analysis for variant discrimination. Temperature melting shift primers are utilized to generate allele-specific amplicons of varying sizes, resulting in distinct melting temperatures during dissociation that enable high-resolution SNV genotype determination [78]. Advantages of this methodology include rapid turnaround time, elimination of post-PCR processing steps, suitability for high-throughput applications, reduced contamination risk, and significant cost savings compared to TaqMan due to the use of non-labeled primers [78,79]. The method demonstrates 100% concordance with Sanger sequencing and can accommodate various DNA preparations [78]. Although sufficient, this method has some limitations, which include sensitivity to experimental conditions such as salt concentration, pipetting accuracy, primer design constraints, and DNA concentration standardization requirements [80]. The high GC content of the *APOE* gene region presents challenges in primer design, particularly for rs7412 genotyping, where high-resolution melting analysis may show poor performance and inconsistent results compared to sequencing validation [80]. Additionally, the presence of primer-dimers may complicate melting curve interpretation, and the method requires optimization for specific amplicon lengths and reaction conditions [79].

#### Allele-Specific Real-Time Quantitative Polymerase Chain Reaction (qPCR)

Although these methods above represented a significant technological breakthrough in their time, their application was limited by multi-step protocols, the need for reaction condition optimization, and relatively low throughput, which increased contamination risk and reduced result reproducibility. Additionally, sensitivity and specificity often varied depending on initial sample quality and primer or probe design, while result interpretation required a high degree of expertise, especially for methods based on melting curves [74,75,76,81,82]. Precisely because of these limitations and demands for greater throughput, precision, and reliability, clinical and research diagnostics today rely on more advanced technologies. At the same time, the classical methods have remained an important foundation. The recent COVID-19 pandemic spurred a rapid evolution in qPCR technology, evidenced by a significant increase in the availability of instrumentation, improved affordability and stability of reagents, and an expansion of technological access worldwide. Real-time qPCR technology progress was followed by the development of new generations of allele-specific methods and TaqMan probes that have enabled simpler, faster, and more robust detection of genetic variants, with significantly greater automation, reduced contamination possibility, and better result standardization [79,83]. Real-time qPCR with allele-specific and TaqMan probes today represents the gold standard due to high sensitivity, specificity, speed, and automation possibilities, and its importance was confirmed in the existing literature [84,85].

The method relies on allele-specific oligonucleotide probes labeled with fluorescent reporters (typically FAM and VIC/HEX) and quenchers, which hybridize precisely to target sequences at polymorphic sites rs429358 and rs7412 [84,85]. During PCR amplification, the 5’ nuclease activity of Taq polymerase cleaves perfectly matched probes, separating the reporter from the quencher and generating allele-specific fluorescence signals that are detected in real-time, while mismatched probes are preferentially displaced without chromophore separation [79,83,86]. The primary advantages include exceptional accuracy with genotyping error rates of less than one case per 2000, rapid turnaround times, high sensitivity and specificity exceeding 99%, scalability for high-throughput applications, and complete closed-tube analysis eliminating contamination risks associated with post-PCR handling [85,86]. The method demonstrates remarkable robustness across different DNA sample types and qualities, with minimal interference from primer-dimer artifacts compared to SYBR Green-based assays, and can accommodate various sample preparation methods [79,84]. Notable limitations encompass, although available, relatively expensive dual-labeled probes compared to unlabeled alternatives, the requirement for specialized real-time PCR equipment with multi-channel fluorescence detection capabilities, and the need for multiple probes per SNV, which increases expenses for multiplexing applications [83,85]. Additionally, probe design requires careful optimization to achieve optimal melting temperature differences, specific GC content parameters, and precise positioning of polymorphic sites within the middle third of nucleotide probes [84,85]. Another limitation regards probe specificity that occurs due to its design primarily targeting common alleles prevalent in populations of European ancestry. This focus may reduce its reliability in identifying rare or ethnicity-specific *APOE* variants. Therefore, for studies involving diverse genetic backgrounds or rare variants, more robust and comprehensive approaches like NGS or Sanger sequencing are recommended to ensure accurate genotyping across populations. Such methods provide broader variant detection capabilities and help circumvent biases introduced by probe design limitations [86,87,88]. Despite cost considerations and probe design limitations, widespread clinical adoption has occurred due to the method’s unparalleled reliability, speed, and ease of implementation in automated platforms, making it the preferred choice for large-scale screening, diagnostic workflows, and genetic association studies where reproducibility and accuracy are essential [84,86]. The technology has largely displaced older gel-based and restriction enzyme methods, enabling precise *APOE* status determination for risk stratification in AD and facilitating the integration of pharmacogenetic testing into routine clinical practice, particularly as personalized medicine approaches become increasingly important in neurodegenerative disease management [79,85].

### 3.3. Sanger Sequencing

Sanger sequencing can also be defined as “oldie goldie” standard for *APOE* genotyping and has played a foundational role in understanding *APOE* genetics since its development by Frederick Sanger in the late 1970s [89]. This chain-termination method utilizes DNA polymerase and fluorescently labeled nucleotides to generate DNA fragments of varying lengths, which are then separated by capillary electrophoresis to reveal the precise nucleotide sequence at polymorphic sites [78,90]. The molecular basis relies on the incorporation of modified nucleotides that lack the 3’-hydroxyl group necessary for chain elongation, effectively terminating DNA synthesis at specific positions and creating a ladder of fragments that correspond to each nucleotide position. Advantages of Sanger sequencing include unparalleled accuracy with error rates below 0.001%, the ability to detect novel mutations and rare variants that might be missed by targeted genotyping approaches, comprehensive coverage of the entire *APOE* coding region, and direct visualization of heterozygous positions through overlapping chromatogram peaks [80,91]. The method serves as the definitive validation tool for other genotyping approaches. It can identify complex genetic variations, including insertions, deletions, and previously unreported polymorphisms that may influence APOE function and disease susceptibility. Primary limitations comprise relatively high cost per sample compared to high-throughput methods, longer turnaround times from PCR to results, lower throughput capacity limiting large-scale population studies, requirement for high-quality DNA templates, and the need for specialized equipment and technical expertise. Additionally, the method requires separate reactions for each DNA strand to confirm heterozygous variants, and interpretation of complex chromatograms can be challenging when multiple variants are present in close proximity. Clinical and research applications extend beyond routine *APOE* genotyping to include identification of rare pathogenic variants associated with lipoprotein glomerulopathy, validation of NGS results, characterization of novel *APOE* mutations in familial dysbetalipoproteinemia, and comprehensive genetic screening in research cohorts investigating AD susceptibility. Despite the emergence of faster, more cost-effective methods, Sanger sequencing remains indispensable for confirming ambiguous results, discovering new variants, and providing the highest level of confidence in genetic diagnosis, particularly in clinical settings where accuracy is superior to speed or cost considerations.

### 3.4. New Approaches

Recent advances in *APOE* genotyping have introduced innovative molecular approaches such as NGS, one-pot PCR genotyping, and fiber optic particle plasmon resonance (FOPPR), each offering unique advantages and limitations compared to established TaqMan PCR assays. NGS enables comprehensive detection of both common and rare *APOE* variants, including novel or complex mutations that may be missed by probe-based PCR methods, by massively parallel sequencing of millions of DNA fragments. At the molecular level, NGS involves fragmenting genomic DNA, ligating adaptors, and amplifying these fragments on a flow cell, followed by cyclic sequencing-by-synthesis reactions that detect nucleotide incorporation via fluorescently labeled reversible terminators. This workflow generates high-resolution data not only for the canonical *APOE* SNVs but also for additional regulatory or coding region variants and co-inherited risk alleles in neurodegenerative disease panels [92]. NGS is particularly valuable in research and clinical settings where complete genetic characterization is required, such as in atypical or early-onset cases, or when simultaneous analysis of multiple genes is necessary. Its strengths include high sensitivity, scalability, and the ability to detect structural variants or rare alleles. However, NGS is more expensive and time-consuming than real-time PCR, requires advanced bioinformatics infrastructure, and may generate incidental findings that complicate interpretation [93].

FOPPR, in contrast, is an amplification-free, label-free biosensing technique that uses single-stranded DNA probes immobilized on gold nanoparticles coated onto an optical fiber. When a sample containing the target DNA is introduced, specific hybridization between the probe and target induces a local refractive index change at the nanoparticle surface, detected in real time as a decrease in light intensity due to enhanced plasmon resonance absorption [94]. Significant advantages include rapid, real-time analysis, label-free detection, high sensitivity, and the ability to regenerate sensor fibers for multiple uses. It is highly resistant to contamination, cost-effective for high-throughput screening, and compatible with minimally processed genomic DNA. However, FOPPR requires careful probe design for specificity, may be less effective for very short or structurally complex DNA fragments, and does not provide sequence-level information or detect unknown variants outside the probe-targeted region, an area where NGS excels [93,94].

One-pot PCR genotyping represents a streamlined approach designed to simplify *APOE* genotyping by combining amplification and allele discrimination in a single closed-tube reaction. This method utilizes allele-specific primers and fluorescent probes that enable simultaneous detection of the key *APOE* polymorphisms without the need for multiple separate reactions or post-PCR processing. The molecular basis relies on the specificity of primer binding to target alleles and real-time fluorescence detection, allowing rapid, accurate, and cost-effective genotyping suitable for clinical diagnostics. One-pot PCR reduces hands-on time, minimizes contamination risk, and supports high-throughput workflows, making it an attractive alternative to traditional multi-step PCR methods [95,96].

A recent study developed a non-invasive, rapid, and cost-effective method for *APOE* genotyping relevant to AD, utilizing DNA extraction with magnetic nanoparticles from a buccal swab sample, followed by real-time PCR with TaqMan probes. This protocol achieved 100% concordance with DNA sequencing, demonstrating reliability and suitability for large-scale screening. The patient-friendly collection process requires minimal resources and allows for room-temperature storage, making it especially practical for elderly and cognitively impaired populations [28].

**Figure 2:**
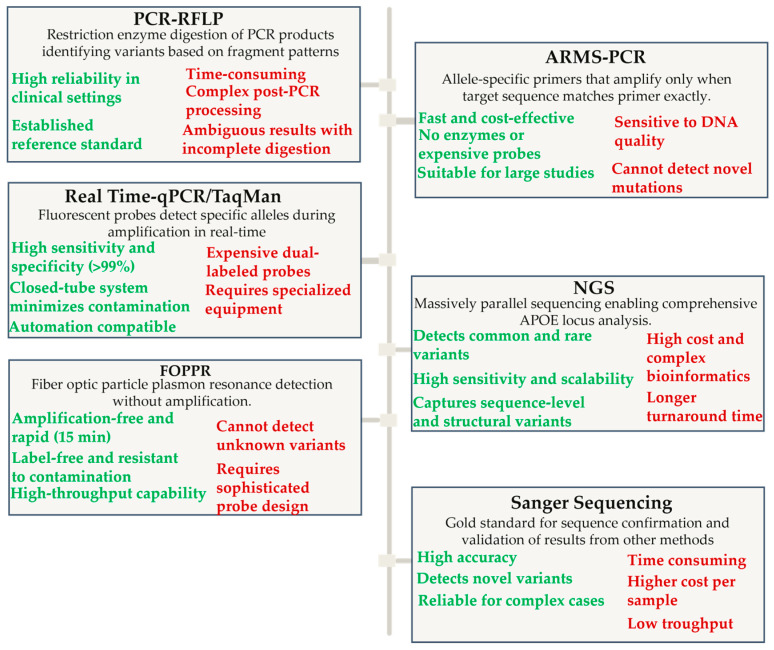
A graphical representation of selected molecular methods for *APOE* status determination. The accompanying scheme succinctly summarizes the key advantages and limitations of each genotyping technique included. Abbreviations: ARMS, amplification refractory mutation system; FOPPR, fiber optic particle plasmon resonance; NGS, next-generation sequencing; PCR, polymerase chain reaction; RFLP, restriction fragment length polymorphism; qPCR, quantitative PCR.

While TaqMan probe-based real-time qPCR remains the clinical standard for routine *APOE* genotyping due to its reliability and cost-effectiveness, NGS, one-pot PCR, and FOPPR are expanding the possibilities for comprehensive and efficient genetic testing. NGS offers unmatched detail and flexibility for variant discovery, one-pot PCR provides rapid and simplified genotyping in a single reaction, and FOPPR offers rapid, simple, and amplification-free genotyping, making these methods attractive for clinical laboratories with high-throughput demands or point-of-care settings. As the clinical importance of *APOE* genotyping grows, especially with the rise of personalized anti-amyloid therapies, the integration of these advanced methods is likely to become increasingly important for optimizing patient care in the era of precision medicine for AD [28,85,95,96]. The long-standing development of methods and approaches for determining *APOE4* status, along with the advantages and limitations of currently available diagnostic techniques, is illustrated in Figure 2.

In this paragraph, an overview of available methods for *APOE* genotyping is provided, focusing on their recommendations and routine use in clinical and research contexts. Real-time PCR with TaqMan probes is routinely recommended for clinical diagnostics due to its high accuracy, speed, and suitability for high-throughput screening, particularly for assessing ARIA risks in anti-amyloid therapies like lecanemab, as endorsed by EMA and CPIC guidelines [97,98]. Sanger sequencing serves as the gold standard for validation and detection of rare variants, but it is not routinely used due to its longer processing time and higher costs [99]. PCR-RFLP, a classical method, is occasionally employed in resource-limited settings for its low cost, but it is less preferred routinely because of potential incomplete digestion and contamination risks [86]. NGS is recommended for research involving comprehensive variant discovery, but it is not routine in clinics owing to high costs and bioinformatics requirements [87]. Overall, method selection should prioritize accuracy, ethical counseling, and alignment with pharmacogenomic guidelines [96].

## 4. Clinical Significance of *APOE4* Status Determination

### 4.1. Risk Assessment and Patient Stratification

Risk stratification based on *APOE* status is being extensively studied, which can influence primary prevention strategies and possible early intervention opportunities [100]. This is particularly relevant in the context of preclinical AD, where individuals are asymptomatic but may already show early pathological changes on positron emission tomography (PET) imaging or in the CSF. For instance, utilizing polygenic risk scores, along with the *APOE4* genotype and astrocytic activation markers, such as glial fibrillary acidic protein (GFAP), and AD biomarkers like phosphorylated tau at threonine 181 (p-tau181), can lead to an increased precision in risk estimates among *APOE4* carriers [101]. Furthermore, *APOE4* is synergistic with other known risk factors for dementia, such as atherosclerosis, traumatic brain injury, type 2 diabetes, and peripheral vascular disease [102,103,104,105].

More precise information is coming, as longitudinal studies, such as the Alzheimer’s Disease Neuroimaging Initiative, have incorporated the *APOE* status in the first and all subsequent studies, and have shown thus far that the *APOE4* genotype predicts both amyloid accumulation and hippocampal atrophy even before the onset of symptoms [106,107]. Furthermore, the FINGER study and the current U.S. POINTER trial have incorporated *APOE* status into analyses of lifestyle interventions, showing that *APOE4* carriers may derive greater cognitive benefit from multimodal prevention programs [108,109,110]. Therefore, genotyping serves both as a predictive marker and a stratification variable for both research and preventive clinical practice.

### 4.2. Association with Earlier Onset, Faster Progression, and Sex Differences

Beyond increasing risk, the *APOE4* allele affects the natural course of AD. Several large-scale cohort studies, including population-based and memory-clinic samples, have demonstrated that *APOE4* carriers develop cognitive symptoms approximately 5–7 years earlier than non-carriers [111]. This is paralleled pathohistologically by earlier and more aggressive accumulation of Aβ plaques and hyperphosphorylated tau tangles, particularly in vulnerable parts of the brain, such as the medial temporal lobe and posterior cingulate cortex [112].

Furthermore, the presence of *APOE4* alleles is also associated with faster progression from mild cognitive impairment (MCI) to dementia, greater rates of brain atrophy on structural magnetic resonance imaging (MRI), and a steeper decline in episodic memory and executive function [113]. These findings are consistent across ethnically and geographically diverse populations, suggesting a robust and reproducible genotype–phenotype link [114].

Finally, there is evidence that sex may modify the impact of *APOE4* on disease risk and progression, as is true for numerous vascular conditions [115]. In contrast to cardiovascular diseases, women develop dementia more often than men, likely due to greater longevity [116]. However, there is also emerging evidence that women who carry the *APOE4* allele have a disproportionately higher risk of conversion to dementia than male carriers, indicating a significant risk factor to consider in the clinic [117]. Hormonal differences, inflammatory response patterns, and brain network connectivity alterations are among the hypothesized mechanisms behind this disparity, although further research is required [118,119,120,121]. In addition, significant interactions have been found between *APOE4* carrier status and use of menopausal hormone therapy for CSF biomarkers. Specifically, *APOE4* carriers who had used hormone therapy exhibited worse levels of CSF p-tau/Aβ_42_ and Aβ_42_/_40_ ratios (where Aβ_42_ and Aβ_40_ represent the 42- and 40-amino acid isoforms of Aβ, respectively) compared to non-users and non-carriers. These findings suggest that the combination of *APOE4* genotype and exogenous hormone exposure may contribute to elevated amyloid burden and AD pathology, particularly in women at increased genetic risk [122]. Finally, there is limited evidence that lifestyle activities are less effective in women who are *APOE4* homozygotes or heterozygotes [123]. These observations underscore the importance of incorporating both sex and *APOE* genotype into predictive models of AD and suggest a need for tailored strategies in diagnosis and management.

### 4.3. Clinical Decision-Making: Should We Genotype All Cognitively Impaired Patients?

Whether *APOE4* status should be routinely determined in patients presenting with cognitive complaints is not yet clear. Genotyping may offer some diagnostic value, particularly in cases with ambiguous clinical features or overlapping pathologies. For example, a positive *APOE4* status may increase the diagnostic probability of AD in a patient with mixed neuroimaging, biomarker profiles, or with assessment with polygenic risk scores. However, it can also be connected with other forms of dementia as well [124]. Still, it must be highlighted that genotyping cannot be used to formulate a diagnosis of the disease, but merely conveys risk, and likely influences treatment or enables better prognostics of disease progression [113,124].

Precision medicine is being introduced in neurodegenerative disease care, and there is a growing number of studies focusing on the genetic part of AD, particularly the *APOE* ε4 allele [125]. As previously established in this manuscript, the *APOE4* carriers differ significantly from non-carriers in terms of disease biology, treatment response, and susceptibility to adverse effects. These differences have implications for both current and emerging therapeutic strategies in AD.

### 4.4. Differential Response to Anti-Amyloid Therapies

The most clinically relevant impact of *APOE4* status to date relates to anti-amyloid monoclonal antibodies. These therapies are being increasingly approved worldwide for AD, with a mechanism of action targeting aggregated Aβ, thereby reducing amyloid plaques and, to a lesser extent, slowing cognitive decline in early-stage AD. However, *APOE4* status influences both efficacy and safety outcomes in these trials and is included in all clinical trials of these therapeutics (Table 1).

*APOE4* carriers are at increased risk of amyloid-related imaging abnormalities (ARIA), particularly vasogenic edema (ARIA-E) and cerebral microhemorrhages (ARIA-H) [126]. Focusing on the trials of approved agents, in the EMERGE and ENGAGE trials of aducanumab, ARIA-E occurred in up to 43% of *APOE4* homozygotes and 35% of heterozygotes, compared to approximately 18% in non-carriers, prompting modified dosing and follow-up protocols [127]. Similarly, in the Clarity AD trial of lecanemab, *APOE4* homozygous carriers had a higher incidence of ARIA-E (32.6%) than non-carriers (5.4%), although efficacy was consistent across genotypes [128]. Similar trends were seen for ARIA-H, with homozygous carriers having a 39,0% incidence, heterozygous carriers 14.0%, and non-carriers having a 11.9% incidence [128]. In the donanemab trial, the incidence of ARIA-E was similar to previous trials, with higher percentages in homozygous (40.6%) and heterozygous carriers (22.8%) compared to non-carriers (15.7%) [129]. In all trials, a significant proportion of symptomatic ARIAs was seen in patients who were homozygous carriers [127,128,129], leading to the aforementioned safety concerns and updated genotyping recommendations.

As a result, *APOE* genotyping is increasingly recommended as part of risk stratification protocols for patients being considered for anti-Aβ therapies. For carriers, particularly homozygotes, the decision to initiate treatment must involve a thorough discussion of ARIA risk, MRI monitoring schedules, and alternative treatment or trial options. Another important consideration is the combination of the *APOE* genotype and anti-Aβ therapies with other comorbidities, as the presence of anticoagulation therapy, the need for thrombolysis, or even antiplatelet therapy can lead to symptomatic ARIA-H, perhaps even in heterozygous carriers [130].

The latest use recommendations for lecanemab state clearly that *APOE* genotyping should be performed for all patients considering the implementation of therapy, given the previously mentioned increased risk [131]. However, the current practice guidelines state that any genetic testing should be preceded by genetic counseling, primarily as the test does not provide definitive diagnostic information [132]. This limitation is compounded by the fact that there is a severe lack of clinical geneticists in most parts of the world [133]. Studies show that knowledge of *APOE4* status can cause anxiety, depression, or changes in self-perception, even in healthy individuals. Protocols developed within the REVEAL studies (Risk Evaluation and Education for Alzheimer’s Disease) have shown that a structured counseling approach can minimize adverse psychological effects [69]. Thus, clear guidelines on genotyping and counseling must be developed sooner, rather than later.

Additionally, these therapies are expensive, and this is a clear limitation even for APOE genotyping in most of countries in the world. Currently, the cost benefit of therapy is not there when compared to the standard of care with anticholinergic therapy, even though there are differences between the antibodies [134,135]. In contrast, genotyping for APOE is not prohibitively expensive as it is a targeted method, with multiple cost-effective and fast options described in the literature [86,136].

**Table 1:** Clinical trials of anti-amyloid therapies that included APOE genotyping.

Antibody	Trial Name/Phase	ClinicalTrials.gov ID(s)	APOE Role
Trontinemab	Brainshuttle AD (Phase I/IIa)	NCT04639050 [133]	*APOE* genotyping for eligibility, stratification, and ARIA surveillance
Remternetug	Early-stage and Phase III trials	NCT05463731 [137]	*APOE* genotyping to assess ARIA risk and stratify analysis
Lecanemab	CLARITY-AD (Phase III)	NCT03887455 [138]	Stratification by *APOE* ε4 status; ARIA monitoring; regulatory guidance on ε4 homozygotes
Gantenerumab	GRADUATE I and II (Phase III)	NCT03444870 [139]	*APOE* ε4 genotyping for safety/ARIA risk; subgroup analyses
	SCarlet RoAD (Phase III)	(Prior trial, published data) [140]	Genotyped for ARIA and cognitive biomarker correlations
Aducanumab	EMERGE and ENGAGE (Phase III)	NCT02484547 [141]	*APOE* ε4 genotyping at screening; higher ARIA incidence in carriers; subgroup analyses
	Post-ARIA safety study	NCT03639987 [142]	Continued dosing with ε4-based ARIA risk profiling
Bapineuzumab [143] (terminated)	Carrier and non-carrier trials (Phase III)	NCT00676143 (carriers), NCT00667810 (non-carriers)	Trials designed separately for ε4 carriers vs. non-carriers; higher ARIA in ε4+ participants
	Long-term extensions	NCT00998764, NCT00606476	*APOE*-based subgroup safety monitoring (ARIA, vasogenic edema)
Crenezumab [144] (terminated)	API Colombia (Preclinical)	NCT01998841	*APOE* and *PSEN1* genotyping; stratification and ARIA risk monitoring
	CREAD (Phase III)	NCT02670083	*APOE*-based inclusion and MRI/ARIA safety surveillance
	Open-label extension	NCT03491150	Continuation of genotyping and monitoring
Solanezumab [145] (terminated)	EXPEDITION 1–3 (Phase III)	NCT00905372, NCT00904683, NCT01900665	*APOE* genotyping performed; subgroup analyses in cognitive/biomarker outcomes
	A4 preclinical study	NCT02008357	Stratified/randomized by *APOE* ε4 status; key prevention platform
	Biomarker substudy	NCT01148498	APOE required for CSF/plasma Aβ analyses and stratification

Abbreviations: AD, Alzheimer’s disease; APOE, apolipoprotein E; API, Alzheimer’s Prevention Initiative; ARIA, amyloid-related imaging abnormalities; Aβ, amyloid beta; CSF, cerebrospinal fluid; MRI, magnetic resonance imaging; NCT, National Clinical Trial (identifier from ClinicalTrials.gov); PSEN1, Presenilin 1 (familial AD mutation gene).

### 4.5. APOE4 Specific Mechanistic Targets and Novel Interventions

Beyond amyloid-centric therapies, *APOE4* carriers may benefit from emerging treatments targeting genotype-specific mechanisms. Due to the known mechanistic effects of APOE4 in AD, targeting focused on it specifically via multiple routes has potential in the future (Table 2) [114].

#### 4.5.1. Antisense Oligonucleotides (ASOs)

ASOs are short, synthetic nucleotides that bind to the mRNA of *APOE*, leading to its degradation or inhibition of translation [146]. In experimental studies using P301S/*APOE4* transgenic mice, ASOs reduced *APOE* expression by nearly 50%, significantly attenuating tau pathology, reducing microglial activation, and improving neuronal survival [147,148]. The possible mechanism is true mitigation of tau aggregation, which, in turn, decreases microglia activation, as measured by decreased microglia staining and lowered inflammatory cytokines like tumor necrosis factor alpha (TNF-α) [144]. Furthermore, it seems that, in general, *E4*-expressing microglia have a higher innate immune reactivity when challenged with lipopolysaccharide, which indicates increased proinflammatory capabilities in this genotype [143]. Despite these promising preclinical results, there are currently no ongoing human clinical trials investigating ASO-based therapies targeting *APOE*.

#### 4.5.2. Gene Editing and Epigenetic Silencing

Clustered regularly interspaced short palindromic repeats/associated protein 9 (CRISPR/Cas9) technology can be used to generate *APOE4* disease models and also test the effects of *APOE* variants, currently in in vitro settings [149]. An example of this is induced pluripotent stem cells-derived organoid induced with a protective *APOE3* variant in the setting of a confirmed *PSEN1* pathogenic variant, based on a case report, which ultimately led to a decrease in p-tau while uncovering novel mechanisms that could be a basis for future therapies [150,151]. Furthermore, a novel concept of in vivo editing was described in a preprint, which focused on editing the ε4 allele to ε3 and succeeded in a small percentage of cells [152]. While still at the experimental stage, these technologies offer the potential for long-lasting allele-specific interventions in the future.

#### 4.5.3. *APOE2* Gene Therapy (LX1001)

An alternative strategy aims to offset *APOE4* toxicity by delivering the protective *APOE2* isoform to the brain using adeno-associated virus (AAV)-based gene therapy. LX1001, developed by Lexeo Therapeutics, is currently being evaluated in a Phase 1/2 open-label trial (NCT03634007) in individuals homozygous for *APOE4*. Interim results have demonstrated increased CSF APOE2 levels, reductions in total tau and phosphorylated tau biomarkers, and a safety profile with no ARIA events reported [153,154]. While promising, it needs to be highlighted that this was a trial without a placebo and that a more extensive randomized controlled trial is necessary to draw any conclusions.

#### 4.5.4. APOE4-Specific Monoclonal Antibodies

Monoclonal antibodies targeting APOE4 offer a selective approach to modulating its pathogenic effects, intending to avoid the vasculature damage that is present in anti-Aβ therapies. The HAE-4 antibody, developed at Washington University, binds aggregated APOE found in amyloid plaques and cerebral amyloid angiopathy in *APOE4*-expressing mice. Treatment with HAE-4 cleared APOE-enriched amyloid deposits and restored cerebrovascular function without increasing microhemorrhages, distinguishing it from anti-Aβ therapies [155].

The Christchurch variant (R136S) of the *APOE3* can provide resistance to AD pathology and symptoms, likely due to its reduced interactions with heparan sulfate proteoglycans (HSPG), ubiquitous cell surface and extracellular matrix components that facilitate Aβ aggregation, cellular internalization, and tau spreading [156]. Since APOE is a critical mediator of HSPG-dependent neurotoxic pathways, this was the basis for the 7C11 antibody, which was designed to disrupt the heparin–APOE4 interactions. Early trials have shown that the antibody reduced the toxicity and tau phosphorylation linked to APOE [156].

#### 4.5.5. Small Molecule and Protein Modifiers

Numerous small molecules are being investigated that focus on the APOE pathway, most of them in the preclinical stage. Recently published data of ALZ-801/Valitramiprosate from the Phase II and Phase III trials show that there is a potential neuroprotective effect of the treatment [157], which is based on disrupting the APP. Importantly, this therapy avoids disrupting the vasculature and appears to be safer in *APOE4* homozygotes and heterozygotes [158,159]. Even though the APOLLOE4 study did not meet the primary endpoint of cognitive improvement, there is a positive statistical signal for patients in the early stage of the disease, and that is promising for the future [157].

Despite promising preclinical data, translation into clinical practice remains limited. Current barriers include fundamental differences between animal models and human disease biology, variability in therapeutic response, and the lack of reliable biomarkers for patient stratification. Moreover, preclinical studies often involve controlled conditions that do not reflect the heterogeneity of human populations, comorbidities, or long-term treatment effects. Regulatory and ethical challenges, as well as the high costs of large-scale clinical validation, further restrict the implementation of novel therapies. These limitations underscore the need for carefully designed translational studies bridging preclinical findings with real-world clinical applicability.

**Table 2:** APOE targeted therapy for AD.

Agent	Modality	Status	APOE4 Mechanism
Anti-APOE ASO	Antisense oligonucleotide	Preclinical	Reduces APOE4 expression; attenuates tau and neuroinflammation
CRISPR/dCas9	Genome/epigenome editing	Preclinical	Converts or silences the *APOE4* allele [151,160]
LX1001	AAV gene therapy	Phase 1/2 (NCT03634007)	Introduces APOE2 to counteract APOE4 toxicity [153]
HAE-4	Monoclonal antibody	Preclinical	Clears APOE-enriched plaques; preserves vascular function [155]
Remternetug	Early-stage and Phase III trials	NCT05463731 [137]	*APOE* genotyping to assess ARIA risk and stratify analysis
7C11	Monoclonal antibody	Preclinical	Disrupts APOE-heparin interactions; prevents neurotoxic conformations [156]
Valitramiprosoate (ALZ-801)	Small molecule (amyloid precursor protein antagonist)	Phase 3	Amyloid precursor protein antagonist, no vasculature adverse effects [159]
Solanezumab [145] (terminated)	EXPEDITION 1–3 (Phase III)	NCT00905372, NCT00904683, NCT01900665	*APOE* genotyping performed; subgroup analyses in cognitive/biomarker outcomes
	A4 preclinical study	NCT02008357	Stratified/randomized by *APOE* ε4 status; key prevention platform
	Biomarker substudy	NCT01148498	APOE required for CSF/plasma Aβ analyses and stratification

Abbreviations: Aβ, amyloid beta; ASO, antisense oligonucleotide; AAV, adeno-associated viral vector; CRISPR/dCas9, clustered regularly interspaced short palindromic repeats/deactivated associated protein 9.

### 4.6. Implications for Lifestyle Interventions and Prevention

Even though we often focus first on pharmacological interventions, there is also compelling evidence that lifestyle interventions may exert differential effects in *APOE4* homozygous and heterozygous carriers.

For instance, *APOE4* carriers show greater vulnerability to cardiovascular and metabolic insults, suggesting they may derive greater benefit from interventions that improve vascular health [161]. Evidence is accumulating that the effects of exercise are different depending on the *APOE* genotype, due to impaired barrier and glucose metabolism. Aerobic exercise, which is a known beneficial factor in AD, did not affect brain-derived neurotrophic factor levels in *APOE4* carriers compared to non-carriers [162], indicating a lesser response. Similar was true for reduced sirtuin-1 levels and baseline insulin signaling compared to the *APOE3* carriers. Given sirtuin-1’s central role in mitochondrial biogenesis, synaptic plasticity, and neuroinflammation, as well as insulin signaling involvement in neuronal survival and cognitive function, these findings imply that *APOE4* carriers may require more intensive or targeted exercise interventions to achieve comparable benefits [163]. A difference in exercise engagement was seen in mice, where *APOE4* mice engaged in lower levels of voluntary wheel-running compared to *APOE3* mice [164]. However, a recent meta-analysis has shown that exercise interventions benefit both *APOE4* carriers and non-carriers equally, even though this conclusion was limited by low-quality evidence [165].

There is evidence of a differential effect in diets about the *APOE4* carriership status. Adherence to a Mediterranean-style diet, rich in polyunsaturated fatty acids and antioxidants, is associated with delayed cognitive decline in *APOE4* carriers [166]. Importantly, individuals with diets containing a large amount of saturated fats showed an increase in cognitive decline, which was not observed in *APOE4* non-carriers [166]. The possible mechanism is due to the differences in the BBB, which is especially pronounced in the medial temporal lobe, a key locus in AD [167].

Finally, sleep quality and stress reduction also play a role. Disrupted sleep and chronic stress exacerbate tau phosphorylation and glymphatic dysfunction, processes particularly active in *APOE4* carriers [168]. Ongoing trials are examining whether sleep optimization and mindfulness interventions have genotype-specific cognitive benefits, although early findings suggest that these interventions may be more effective in *APOE4* carriers compared to non-carriers [110,123,169].

Taken together, these findings underscore the growing importance of integrating *APOE4* genotyping into personalized prevention strategies, particularly for individuals in the early stages of AD. While no single intervention has proven disease-modifying effects in *APOE4* carriers, the combination of lifestyle optimization and genotype-informed pharmacotherapy may offer a promising pathway to delay onset and slow progression.

## 5. Early Biomarkers and Monitoring APOE4 Carriers

The diagnosis and staging of AD have been addressed in numerous clinical guidelines, all of which emphasize the integration of biomarker data with clinical evaluation. AD is now recognized as a biological disease entity that can be diagnosed in vivo based on the presence of specific biomarkers, regardless of whether clinical symptoms are present [170,171]. This paradigm shift enables the detection of AD pathology even in cognitively unimpaired individuals, thereby opening avenues for earlier and potentially more effective intervention.

A variety of laboratory and imaging biomarkers are available to serve as surrogate indicators of the underlying disease processes across the clinical spectrum, from asymptomatic stages to MCI and dementia. These biomarkers have been incorporated into the biological framework of AD and categorized using the ATN-I classification system, which groups them by Aβ deposition (A), pathological tau (T), neurodegeneration (N), and neuroinflammation (I). Neurodegenerative alterations, which ultimately lead to dementia owing to AD, occur about 20 years before the appearance of clinical symptoms [172].

In clinical trials, Aβ pathology is typically confirmed through PET imaging or by assessing the Aβ in CSF. CSF biomarkers, including Aβ_42_, the Aβ_42_/_40_ ratio, and p-tau181, are among the most reliable indicators of AD pathology. Decreased CSF Aβ_42_ and Aβ_42_/_40_ ratio reflect early amyloid plaque deposition and correlate negatively with amyloid PET imaging. Notably, the Aβ_42_/_40_ ratio more accurately predicts amyloid PET positivity than Aβ_42_ alone. Elevated CSF p-tau181 reflects tau pathology and, when combined with Aβ_42_ as a ratio, predicts cognitive decline and conversion to AD dementia [128,173]. These biomarkers not only precede clinical symptoms but also aid in differential diagnosis [174], making them valuable tools in understanding genetic risk factors such as APOE and evaluating their contribution to AD pathology.

Many studies have assessed the impact of *APOE* on these biomarker trajectories. Human PET imaging studies have demonstrated that, among cognitively normal individuals, Aβ deposition is the highest in those with the *APOE4/E4* genotype, with the pattern of amyloid accumulation consistently following the order: *APOE4/E  *> *APOE4/E3* > *APOE3/E3* > *APOE2/E3* across all age groups [175,176]. Consistent with these human findings, mouse models engineered to develop cerebral Aβ pathology exhibit a similar genotype-dependent pattern in both onset and extent of amyloid deposition, with the order being *E4* > *E3* > *E2* [176,177]. Furthermore, accumulating evidence suggests that amyloid-related tau abnormalities progress more rapidly in *APOE4* carriers, suggesting that *APOE4* may facilitate earlier and more extensive amyloid-driven tau propagation across functionally connected brain regions [178]. Animal studies using tau transgenic models have shown that *APOE4* exacerbates tau pathology and neurodegeneration, with severity following the order *E4* > *E3* > *E2 > APOE* knockout. Notably, partial reduction in *APOE4* expression, either globally or in specific cell types like astrocytes or neurons, significantly attenuates tauopathy, neuroinflammation, synaptic loss, and brain atrophy, highlighting the therapeutic potential of *APOE4* silencing in tau-driven neurodegeneration [43,55,147].

Apart from Aβ and tau biomarkers, blood-based indicators of neurodegeneration in AD include total tau, GFAP, and neurofilament light chain (NfL), reflecting neuronal injury, CNS inflammation, and subcortical axonal damage, respectively. These biomarkers reliably predict future cognitive decline, often years before clinical symptoms emerge [179,180]. Significantly, as shown in human and animal studies, the *APOE4* allele modulates both the strength and direction of these associations, underscoring a gene-dependent susceptibility to neurodegeneration [147,181]. Findings from Fortea et al. [21] revealed that nearly all *APOE4* homozygotes presented with AD pathology and increased concentrations of both CSF (Aβ1–42 and p-tau) and plasma biomarkers (p-tau and NfL), supporting the use of *APOE4* as a proxy for AD pathophysiology. Notably, in a 20-year longitudinal study involving a diverse cohort of older adults without dementia, *APOE4* carrier status was shown to significantly accelerate cognitive decline in the presence of elevated blood-based biomarkers of neurodegeneration (total tau, NfL, GFAP) [182].

Altogether, these insights emphasize the critical role of *APOE* genotyping not only in understanding biomarker dynamics but also in identifying subgroups who may benefit from more tailored preventive and therapeutic strategies. Incorporating *APOE* genotype into biomarker-based definitions of AD could enhance both clinical decision-making and trial design in the era of precision medicine. To support these efforts, it would also be beneficial to develop APOE-related biomarkers, such as neuron-derived APOE4 fragments or APOE4-induced molecules (e.g., major histocompatibility complex class 1, high mobility group box 1) [48] measurable in CSF or plasma, which could enable improved patient stratification and monitoring of APOE4-targeted therapies.

## 6. Conclusions and Future Directions

As AD-related dementia remains incurable, current research has increasingly focused on the prodromal and preclinical stages, where biomarker-based detection may enable earlier diagnosis and offer a critical window for therapeutic intervention. In addition to fluid and imaging biomarkers, genetic markers such as the *APOE4* allele have emerged as pivotal tools in AD risk stratification, early detection, and research-based screening. Although *APOE* genotyping is not yet part of standard clinical diagnostic protocols, its incorporation provides valuable prognostic insights, particularly in preclinical and prodromal stages, by refining individual risk profiles. New insights that *APOE4* homozygosity represents a distinct genetic form of AD reinforce the clinical value of routine *APOE* genotyping not only for risk stratification but also for aligning individuals, particularly *APOE4* homozygotes, with personalized monitoring and intervention strategies reflective of their genetically driven disease trajectory. Integrating *APOE* status with established biomarker frameworks, such as the ATN-I classification, could significantly enhance the precision of patient stratification in clinical trials and guide personalized preventive and therapeutic strategies.

Mounting evidence supports a toxic gain-of-function role of APOE4 in AD pathogenesis, with studies in preclinical models consistently demonstrating that reducing APOE4 levels alleviates multiple pathological features, including amyloid and tau pathology, neurodegeneration, and neuroinflammation. These findings underscore the need to prioritize the development of APOE4-targeted therapies. Routine *APOE* genotyping could further facilitate early and individualized interventions, optimizing both prevention and treatment decisions.

Moreover, gene–environment interactions, such as those observed between *APOE4* status and menopausal hormone therapy, have been shown to exacerbate AD biomarker abnormalities in genetically at-risk individuals. Clinical implications extend to treatment safety as well. The incidence of ARIA, a known adverse effect of anti-amyloid monoclonal antibodies, is significantly higher in *APOE4* carriers. This highlights the potential of *APOE* genotyping not only for stratifying therapeutic benefit but also for mitigating treatment-related risks. Given these considerations, a combinatorial therapeutic approach that includes APOE4-lowering strategies alongside existing anti-amyloid therapies may offer enhanced efficacy while minimizing adverse effects in genetically susceptible individuals.

While *APOE* is most extensively studied in the context of AD, its broader involvement in other neurological and cardiovascular disorders further underscores its relevance in precision medicine. In this context, various *APOE* genotyping methods, including Sanger sequencing, real-time qPCR, RFLP-PCR, and newer platforms like one-pot PCR, NGS, and FOPPR, differ in their sensitivity, scalability, and clinical utility. While traditional methods retain value due to their accuracy and accessibility, emerging technologies hold promise for high-throughput, minimally invasive, and point-of-care applications. Future research should aim to optimize and integrate these platforms, particularly one-pot PCR and FOPPR-based biosensing, into clinical workflows to enable rapid, cost-effective, and personalized detection of *APOE4* alleles.

It is important to note that *APOE4* genotyping carries important ethical challenges, including the need for thorough informed consent and genetic counseling due to the limited predictive value of the test and possible psychological consequences. Genetic counseling before and after testing represents a key component of a responsible approach. Moreover, although *APOE* genotyping is relatively inexpensive and technically feasible, its broader implementation is constrained by cost-effectiveness considerations, healthcare infrastructure, and issues of equitable access, particularly in resource-limited settings. Taken together, these considerations suggest that while *APOE* genotyping holds promise as a cornerstone of individualized, mechanism-based interventions in AD, its integration into routine practice must be pursued cautiously and context-sensitively.

## Abbreviations

The following abbreviations are used in this manuscript (Note: Some abbreviations (e.g., trial names and protocols) refer to specific clinical trials as outlined in the text.):

AD	Alzheimer’s disease
Aβ	Amyloid beta
APP	Amyloid precursor protein
API	Alzheimer’s Prevention Initiative
APOE	Apolipoprotein E
APOE2	Apolipoprotein E, epsilon 2 allele
APOE3	Apolipoprotein E, epsilon 3 allele
APOE4	Apolipoprotein E, epsilon 4 allele
ARIA	Amyloid-related imaging abnormalities
ARIA-E	Amyloid-related imaging abnormalities vasogenic edema
ARIA-H	Amyloid-related imaging abnormalities cerebral microhemorrhages
ARMS	Amplification refractory mutation system
ASO	Antisense oligonucleotide
AAV	Adeno-associated virus
BBB	Blood-brain barrier
CRISPR/Cas 9	Clustered regularly interspaced short palindromic repeats/associated protein 9
CNS	Central nervous system
CSF	Cerebrospinal fluid
FOPPR	Fiber optic particle plasmon resonance
GFAP	Glial fibrillary acidic protein
HSPG	Heparan sulfate proteoglycans
MCI	Mild cognitive impairment
MRI	Magnetic resonance imaging
NfL	Neurofilament light chain
NGS	Next-generation sequencing
PCR	Polymerase chain reaction
p-tau	Phospho tau
PET	Positron emission tomography
PSEN1	Presenilin 1
qPCR	Quantitative polymerase chain reaction
RFLP	Restriction fragment length polymorphism
SSP-PCR	Sequence-specific primer polymerase chain reaction
